# Evaluating the medical direct costs associated with prematurity during the initial hospitalization in Rwanda: a prevalence based cost of illness study

**DOI:** 10.1186/s12913-022-08283-w

**Published:** 2022-07-27

**Authors:** Anaclet Ngabonzima, Domina Asingizwe, David Cechetto, Gisele Mukunde, Alain Nyalihama, Mathias Gakwerere, David Mark Epstein

**Affiliations:** 1 JSI Research & Training Institute, Inc, Washington, DC USA; 2 College of Medicine and Health Sciences, University of Rwanda, Kigali, Rwanda; 3 Schulich School of Medicine & Dentistry, University of Western Ontario, London, ON Canada; 4 PIVOT Works Inc, Ranomafana, Madagascar; 5 United Nations Population Fund (UNFPA), Kigali, Rwanda; 6 University of Granada, Granada, Spain

**Keywords:** Prematurity, Preterm birth, Medical direct cost

## Abstract

**Background:**

Prematurity is still the leading cause of global neonatal mortality, Rwanda included, even though advanced medical technology has improved survival. Initial hospitalization of premature babies (PBs) is associated with high costs which have an impact on Rwanda’s health budget. In Rwanda, these costs are not known, while knowing them would allow better planning, hence the purpose and motivation for this research.

**Methods:**

This was a prospective cost of illness study using a prevalence approach conducted in 5 hospitals (University Teaching Hospital of Butare, Gisenyi, Masaka, Muhima, and Ruhengeri). It included PBs admitted from June to July 2021 followed up prospectively to determine the medical direct costs (MDC) by enumerating the cost of all inputs. Descriptive analyses and ordinary least squares regression were used to illustrate factors associated with and predictive of mean cost. The significance level was set at *p* < 0.05.

**Results:**

A total of 123 PBs were included. Very preterm and moderate PBs were 36.6% and 23.6% respectively and the average birth weight (BW) was 1724 g (SD: 408.1 g). The overall mean MDC was $237.7 per PB (SD: $294.9) representing 28% of Gross Domestic Product (GDP) per capita per year. Costs per PB varied with weight category, prematurity degree, hospital level, and length of stay (LoS) among other variables. MDC was dominated by drugs and supplies (65%) with oxygen being an influential driver of MDC accounting for 38.4% of total MDC. Birth weight, oxygen therapy, and hospital level were significant MDC predictive factors.

**Conclusion:**

This study provides an in-depth understanding of MDC of initial hospitalization of PBs in Rwanda. It also indicates predictive factors, including birth weight, which can be managed through measures to prevent or delay preterm birth.

**Implication for prematurity prevention and management:**

The results suggest a need to revise the benefits and entitlements of insured people to include drugs and interventions not covered that are essential and where there are no alternatives. Having oxygen plants in hospitals may reduce oxygen-related costs. Furthermore, interventions to reduce prematurity should be evaluated using cost-effectiveness analysis since its overall burden is high.

**Supplementary Information:**

The online version contains supplementary material available at 10.1186/s12913-022-08283-w.

## Background

Preterm or premature babies (PBs) are those born before 37 weeks of gestation. Though all newborns are vulnerable, PBs are acutely so because some of its organs are not fully developed at birth [1]. From a clinical point of view, preterm birth (PTB) is classified from extremely (< 28 weeks), very (28 to < 32 weeks), moderate (32 to < 34 weeks) to late preterm (34 to < 37 weeks) [1]. Worldwide, 15 million PBs are born every year indicative of a preterm birth rate of 11% and over 84% of them occur at 32–36 weeks of gestation [1, 2]. Currently, prematurity is the leading cause of both neonatal and child death worldwide [3]. Sub-Saharan Africa and South Asia account for over 60% of preterm birth worldwide [1]. In Rwanda, recent studies showed that the PTB rate is estimated at around 10% and that prematurity is the primary risk factor for neonatal and child mortality countrywide [4, 5]. It is worth mentioning that prematurity is frequently associated with low birth weight (weight < 2500 g at birth) which is also a major contributor to newborn mortality [5, 6].

After birth, initial management of a premature baby requires hospitalization which is associated with diagnostic and inpatient costs with most early premature babies requiring admission to the intensive care unit (ICU) for an extended period of time [7, 8]. Although preterm birth is still the leading cause of neonatal and child mortality in developing countries, the chances of survival of PBs, including even very preterm babies, have increased with recent advances in medical technology. This improvement in survival is associated with an increase in length of stay (LoS) resulting in increased hospitalization-related costs. PTB costs constitute a large portion of hospital budgets worldwide and are considered among the most costly hospital admissions and account for a high proportion of total costs in any neonatal unit [6–9]. Finally, depending on the health system, there can also be a substantial, even catastrophic, financial burden on families [6–11]. Therefore, evaluating the cost of PB is particularly crucial as resources are scarce and each expenditure has a cost-benefit. In addition, analyzing the costs associated with initial hospitalization of PBs can allow countries to consider the alternatives including promoting measures to prevent or delay premature delivery among others.

Studies have evaluated the costs and drivers of costs associated with initial hospitalization of premature babies in upper middle income, developed, and developing countries. In Brazil, Desgualdo et al. in 2004 revealed that the average direct cost of care for the initial hospitalization of a premature baby was $2386 and that the daily average cost of a premature baby weighing less than 1000 g was $115. Costs were dominated by diagnostic testing-related costs [12]. In Malaysia, Zainal et al. showed that the median cost for initial hospitalization of a premature baby ranged from $1722 ($ 111 per day) for late preterm to $11,532 ($ 135 per day) for extreme preterm [13]. In a study conducted in Turkey by Comert et al., the mean total hospitalization cost of a premature baby was $4187 ($303 per day) [14]. In Greece, Geitona et al. found an average cost per premature baby of $6801 [15] while Akman et al. in Turkey reported the cost per premature baby of $4345 ($ 250 per day) [16]. In India, Naranga et al. calculated $125 as the mean cost per day of hospitalization [17]. In Nigeria, a study conducted to evaluate the economic burden of preterm/very low birth weight care revealed that the direct cost of care ranged from US$80 to $1055 with a median of $247.3 [18].

Although measuring the medical direct costs (MDC) of initial hospitalization for premature babies is important to guide the country’s public health policies, strategies, and budget setting to tackle preterm birth-related morbidity and mortality, this has not been adequately investigated in many low and lower-middle-income countries [4, 5]. Likewise, in Rwanda, despite the high preterm birth (PTB) rate and morbidity associated with this condition [5], no study has been published on the costs of initial hospitalization of premature babies and therefore, the related cost predictors are not known. Thus, the results of this research will address this gap and inform better planning and prioritization of interventions aimed at caring for and treating this condition in Rwanda as well as those aimed at reducing its incidence.

## Methods

### Study area

The study was carried out in 5 hospitals in Rwanda namely the University Teaching Hospital of Butare (UTHB), Gisenyi district hospital, Masaka district hospital, Muhima hospital, and Ruhengeri referral hospital. The choice of these hospitals was two-fold: first, they present the different levels of care with UTHB being a tertiary hospital, Ruhengeri a regional referral hospital, Masaka and Gisenyi are district hospitals while Muhima is considered to be a center of excellence in neonatal care even though it is also considered to be a district hospital. Secondly, they are among hospitals admitting a higher number of PBs in the country.

### Study design and approach

This was a prospective economic study, a cost of illness (COI) type using the prevalence approach as the costs were allocated to the year in which they occurred. The study was conducted from the healthcare payer perspective. We used the micro-costing (bottom-up) approach since the cost estimation method involved the direct enumeration and costing out of every input consumed for the management of each PB included in this study [19, 20].

### Study population and sample

The study population included all PBs admitted to neonatal units of the five hospitals and their mothers during the study period (June–July 2021). All babies born before reaching 37 weeks of gestation and admitted to the neonatal units during the study period and meeting inclusion criteria were included. Each case was followed prospectively over the period of initial hospitalization to determine the associated MDC incurred. Mothers of the newborns included in this study were also interviewed to provide information regarding their socioeconomic and demographic characteristics.

### Exclusion criteria

All premature babies admitted for less than 24 hours and those admitted with severe congenital abnormalities to receive only supportive care due to their short duration of life were automatically excluded. The latter were excluded as we considered it unethical to inform the mother that the study aims to determine the cost of managing the newborn when the poor outcome is already known.

### Data collection instrument

A data collection tool was developed and tested prior to data collection. MDC included costs related to consultation, clinical follow-up, lab investigations, imaging, hospitalization, drugs and supplies, phototherapy, enteral and parenteral nutrition, infusions, professional services, and other management-related fees qualified as a direct cost. Data collection was conducted in all services of the neonatal unit of the concerned hospitals. Unit costs were obtained from the list established by the Ministry of Health (MOH) and health insurances [21]. Where the unit cost was not on the list, the prices of the central medical store were applied. Other variables were also collected to inform the predictive model. Those included clinical information of the mother, baby anthropometric measures, length of stay (LoS), and gender. The Appearance, Pulse, Grimace, Activity, Respiration (APGAR) score was collected which is a measure of newborn ‘physical condition’ obtained by adding points (2, 1, or 0) for heart rate, respiratory effort, muscle tone, response to stimulation, and skin coloration; a score of ten representing the best possible condition [22]. These instruments have been validated in previous studies [4, 6–10]. The outcome variable was the total MDC per PB. The mothers’ socioeconomic class was measured according to “*Ubudehe system*”. *Ubudehe system* is a social stratification programme depending on income among households using categories. These categories range from 1(lowest) to 4 (highest) socioeconomic classes (https://rwandapedia.rw/hgs/ubudehe/poverty-level), [23].

### Data collection

Research approvals were obtained from the Institutional Review Board (IRB) of the College of Medicine and Health Sciences, the University of Rwanda (Approval Notice: No 176/CMHS IRB/2021), and the Rwanda National Ethics Committee (RNEC) (Review Approval Notice: No.580/RNEC/2021). A copy of the research questionnaire along with copies of the research approvals were sent to each hospital to request permission to conduct the study. A researcher at each hospital was trained to help the Principal Investigator (PI) in data collection. When permission was formally granted by the hospital, the data collection started. Data collection for MDC incurred was done by data collectors by enumerating the cost of all inputs using medical files on a daily basis until the PB was discharged from the hospital. Mothers of premature babies who met the inclusion criteria and agreed to participate in the study were given a consent form to sign voluntarily. These mothers were interviewed to provide the information regarding their socioeconomic and demographic characteristics and this was done once at any time when clinical conditions were stable.

### Statistical analysis

All cost-related data were reported in Rwandan Francs (RWF) and American Dollars where US$1 = RWF 980 (prevailing conversion rate in mid-2021). Data was tabulated with Microsoft Excel (2016) and subsequently exported into SPSS 25 for further analysis. Descriptive analysis was performed for continuous and categorical variables. Average medical direct cost (MDC) per PB was determined by summing up the total MDC obtained through micro-costing of each PB and dividing it by the number of PBs in the study. Results were also stratified by different study variables. In addition, MDC per day per PB was calculated. The total MDC of initial hospitalization of a PB in Rwanda was estimated based on the recent preterm birth incidence [4, 5]. Linear regression by ordinary least squares was used to identify predictive factors of MDC. The significance level was set at *p* < 0.05. We ensured that related independent variables were not included in the same model by performing a collinearity test. In addition, the coefficient of determination (R-squared) was calculated to measure how much of the variation in outcome can be explained by the variation in the predictor(s).

## Results

### Characteristics of premature babies included in the study

The study included 123 PBs meeting the inclusion criteria from five study hospitals. As per Table 1, very premature (28 to< 32 weeks) and late PBs (34 weeks to < 37 weeks) were 36.6 and 39.8% respectively of the total sample. Very low birth weight (1000 to < 1500 g) and moderate low birth weight babies (1500 to < 2500 g) were 34.1 and 62.6% respectively. The majority (78.4%) were born to mothers who had not taken corticosteroids when indicated, i.e. less than 34 weeks of gestation. Most of the deliveries (89.4%) had taken place in the health facility.

**Table 1:** Characteristics of preterm births per category

Variables	Variable category	Frequency	Percent
**Sex**	Male	59	48.0
	Female	64	52.0
	**Total**	**123**	**100.0**
**Age in weeks**	28 to < 32	45	36.6
	32 to < 34	29	23.6
	34 to < 37	49	39.8
	**Total**	**123**	**100.0**
**Weight in grams**	< 1000	2	1.6
	1000 to < 1500	42	34.1
	1500 g to < 2500	77	62.6
	2500 and more	2	1.6
	**Total**	**123**	**100.0**
**Mothers took corticosteroids if indicated**	Yes	16	21.6
	No	58	78.4
	**Total**	**74**	**100.0**
**APGAR Score at 5th minute**	< 4	2	1.9
	4 to 6	4	3.7
	7 and more	102	94.4
	**Total**	**108**	**100.0**
**Place of birth**	That hospital	90	73.1
	Other health facility	20	16.3
	Home	13	10.6
	**Total**	**123**	**100.0**
**Hospital study**	Gisenyi	25	20.3
	Masaka	25	20.3
	Muhima	25	20.3
	UTHB	25	20.3
	Ruhengeri	23	18.8
	**Total**	**123**	**100.0**

As per supplementary file 1, the average LoS was 14.7 days (SD: 13.5) with a minimum and maximum of 2 and 67 days respectively. The highest and lowest LoS were registered in regional referral and district hospitals respectively. Birth weight (BW) ranged from 900 to 2970 g (SD: 408.1) with an average of 1724 g. The age at birth ranged from 28 weeks to 36 weeks and 5 days with an average of 32 weeks and 3 days and a median of 32 weeks and 5 days.

### Characteristics of mothers

The majority of mothers (67,4%) were in a low socioeconomic class, either category 1 or category 2 of the Ubudehe scale. With regards to insurance, almost all mothers were insured; 92.7% with Community Based Health Insurance Scheme (CBHI). Details are shown in supplementary file 2.

### Clinical features of premature babies and length of stay

As indicated in supplementary file 3, clinical features were dominated by respiratory distress syndrome (60%) and jaundice (53%). Most of the PBs were discharged from hospitals with clinical improvement (86.2%). The majority of premature babies (64.3%) spent less than 15 days in the hospital.

### Medical direct costs analysis

In this section, the medical direct costs are analysed taking into account different variables including types of medical direct costs, hospital level, prematurity level, birth weight, length of stay, sex, outcome, place of birth, mode of delivery, and corticosteroids use.

#### Medical direct costs total and per category

The cost data tables indicate the costs in Rwandan francs (RWF) and US dollars (USD). As shown in Table 2, the total MDC for treating 123 premature babies (PBs) was $29,240 and the average MDC for treating one PB was $237.7. The maximum average MDC was $1569.4.

**Table 2:** Mean medical direct cost per preterm birth and by service

Category	Total costs RWF (US$)	Average RWF (US$)	Maximum RWF (US$)	% of Total
Laboratory tests	2,137,322.0 (2180.9)	17,377 (17.7)	187,725 (189.8)	7.5
Imaging	68,725.7 (70.1 $)	559 (0.5)	24,015 (24.5)	0.2
Drugs & supplies (O2 excluded)	7,610,970.2 (7766.3)	61,878 (69.5)	328,680 (335.3)	26.6
Oxygen	10,990,667.0 (11,215.0)	89,355 (91.1)	829,310 (846.2)	38.4
Human resources services	4,934,081.8 (5034.8)	40,114 (40.9)	402,018 (410.2)	17.2
Hospitalization	2,568,960.0 (2621.4)	20,886 (21.3)	324,000 (330.6)	8.9
Ambulance	344,140.1 (351.2)	2798 (2.8)	75,200 (76.7)	1.2
Total Costs	28,654,866.9 (29,240.0)	232,966 (237.7)	1,538,049 (1569.4)	100

Disaggregating cost per category, the oxygen-related cost amounted to 38.4% of the total MDC. Other drugs & supplies and professional services amounted to 26.6 and 17.2% respectively. In total, all drugs and supplies accounted for about 65% of total MDC.

#### MDC per hospital level, prematurity, preterm birth weight and length of stay

Breaking down the costs by hospital level and prematurity as shown in Table 3, the average MDC for treating a PB admitted to a district hospital was $136.4. The average MDC was $431.4 and $363.2 in regional referral and tertiary hospitals, respectively. Regarding the level of prematurity, the average MDC of treating a very PB was $391.3 and $91.2 for late PB.

**Table 3:** Mean medical direct cost per hospital level, prematurity, birth weight and length of stay

Variable	Category	Average RWF (US$) per PB	Maximum RWF (US$) per PB	Minimum RWF (US$) per PB
**Hospital category**	District hospitals	133,723 (136.4)	1,283,473 (1309.6)	11,598 (11.8)
	Regional referral	422,863 (431.5)	1,396,051 (1424.5)	41,911 (42.7)
	Tertiary	355,990 (363.2)	1,538,049 (1569.4)	85,167 (86.9)
**Level of prematurity**	28 to < 32 weeks	383,544 (391.3)	1,538,049 (1569.4)	11,598 (11.8)
	32 to < 34 weeks	241,864 (246.8)	1,283,473 (1309.6)	17,835.25 ($18.2)
	34 weeks and more	89,414 (91.2)	429,245 (438.0)	14,977 (15.2)
**Birth Weight**	1000 to < 1500	391,402 (399.3)	1,538,049 (1569.4)	33,384 (34.0)
	1500 to < 2500	130,029 (132.6)	929,675 (948.6)	11,598 (11.8)
**LoS**	< 8 days	75,578 (77.1)	303,675 (309.8)	11,598 (11.8)
	8 to < 15 days	161,924 (165.2)	440,759 (449.7)	24,175 (24.6)
	15 to < 22 days	293,621 (299.6)	760,837 (776.3)	87,360 (89.1)
	22 to < 29 days	397,986 (406.1)	1,538,049 (1569.4)	69,527 (70.9)
	> = 29 days	679,942 (693.8)	1,396,051 (1424.5)	57,101 (58.2)

LoS = Length of stay

Considering BW, the average MDC to treat a very low BW baby was $399.3 while it was $132.6 for a low BW one.

Finally, by taking into account the length of stay (LoS), the average cost of treating one PB varies considerably from $77.1 for those admitted for less than 8 days to $693.8 for those who stayed from 29 days upwards. The average MDC per day and per bed is $16.4.

#### MDC per sex, outcome, place of birth, mode of delivery and corticosteroids

As per Table 4, the average MDC per PB discharged alive was $220.6. The average was $491.3 for those referred to another health facility and $312.7 for those who died. The average MDC was $245.7 for a PB born in the study hospital while it was $364.5 for those born in a health center of another hospital catchment area. The average MDC was $261.9 for PB born by normal delivery and $187.4 for those born by C-section. In most of these cases, the average BW and LoS varied considerably.

**Table 4:** MDC per sex, outcome, place of birth, mode of delivery and corticosteroids

Variable	Category	Average RWF (US$)	Maximum RWF (US$)	Minimum RWF (US$)
**Sex**	Female	239,733 (244.6)	1,538,049 (1569.4)	14,977 (15.2)
	Male	225,626 (230.2)	1,283,473 (1309.6)	11,598 (11.8)
**Outcome**	Improved	216,229 (220.6)	1,538,049 (1569.4)	11,598 (11.8)
	Referred	481,485 (491.3)	760,837 (776.3)	171,604 (175.1)
	Died	306,442 (312.7)	654,020 (667.3)	63,100 (64.3)
**Place of birth**	Hospital study	240,867 (245.7)	1,538,049 (1569.4)	11,598 (11.8)
	HC of hospital study	171,017 (174.5)	434,326.40 (443.1)	14,976.54 (15.2)
	HC of another hospital	357,228 (364.5)	760,837 (776.3)	200,777 (204.8)
	Home BW	202,679 (206.8)	1,033,906 (1055.0)	17,835 (18.2)
	In another hospital	229,944 (234.6)	345,304 (352.3)	169,972 (173.4)
**Corticosteroids**	Taken	517,483 (528.0)	1,396,051 (1424.5)	124,841 (127.3)
	Not taken	263,611 (268.9)	1,538,049 (1569.4)	11,598 (11.8)
**Mode of delivery**	Normal delivery	256,734 (261.9)	1,396,051 (1424.5)	11,598 (11.8)
	C section	183,648 (187.4)	1,538,049 (1569.4)	22,123 (22.5)

### Coverage by insurance

Of the 123 PBs, 122 had insurance coverage. As shown by Table 5, in general, 87.0% of total costs incurred by admitted PBs whose mothers are covered by any insurance scheme are part of the covered benefits while 13.0% of the total costs are not. Half of total the costs related to professional services are not covered. A further breakdown revealed that key items not covered include the use of continuous positive airway pressure (CPAP) machine, the time spent in an incubator, vital signs taking, perfusion change, ward rounds, and first consultation by nurses among others. In addition, 15.8% of the total costs incurred in the category of drugs and supplies (oxygen excluded) are not covered. Key drugs and supplies not covered include iron and folic acid supplement, caffeine, aminophylline, and alcohol. All laboratory-related test costs are covered.

**Table 5:** MDC covered versus not covered by insurance

Cost category	Category Total RWF (US$)	Amount covered RWF (US$)	% covered
**Laboratory tests**	2,134,260 (2178)	2,134,260 (2178)	100.0
**Imaging**	68,726 (70)	62,535 (64)	91.0
**Drugs & supplies** (O2 excluded)	7,606,024 (7761)	6,406,533 (6537)	84.2
**Oxygen**	10,985,587 (11,210)	10,985,587 (11,210)	100.0
**Professional services**	4,919,392 (5020)	2,416,217 $2466)	49.1
**Hospitalization**	2,567,040 (2619)	2,567,040 (2619)	100.0
**Ambulance**	344,140 (351)	344,140 (351)	100.0
**Total Costs**	28,625,169 (29,209)	24,916,313 (25,425)	87.0

#### Estimating the total annual medical direct costs

The calculated average MDC for PBs in the 5 hospitals in the study allowed us to make an estimate of health expenditure on PBs hospitalization in the country. Based on the number of births in Rwanda estimated to be around four hundred thousand (400,000) per year [24] and taking into account the recent 10% preterm birth rate [5], we estimated the annual number of PBs to be about 40,000. Furthermore, about 90% of the newborn admissions occur in district hospitals where the average MDC of initial hospitalization is $136.4 and approximately an average of $400 for referral and tertiary hospitals as indicated in Table 6 [25]. Thus, the health expenditure for the initial hospitalization of PBs was estimated to be more than $6,500,000 annually.

**Table 6:** Factors predicting medical direct costs, ordinary least squares

Variables	Beta coef. [RWF]	Std. Error [RWF]	t	***P***-value
(Constant)	312,933.0	267,768.4	1.169	0.247
**Corticosteroid not taken (ref)**				
Corticosteroid taken	95,482.7	83,918.6	1.138	0.260
**District hospital (ref)**				
Regional Referral Hospital	313,127.2	88,846.8	3.52	**0.000**
Tertiary Hospital	165,503.1	89,393.1	1.85	**0.049**
**Insured with CBHI (ref)**				
Insured with RSSB/RAMA	357,473.9	152,152.2	2.35	**0.022**
Insured with other private insurance	−215,405.6	260,137.3	−0.82	0.411
**Born in the hospital study (ref)**				
Born in HC of this hospital CA	−22,780.8	89,032.4	−0.25	0.799
Born in HC of another catchment area	2745.3	128,890.2	0.02	0.983
Born Elsewhere in another facility	47,049.2	192,900.4	0.24	0.808
**No oxygen (ref)**				
Oxygen therapy	228,758.3	86,647.4	2.64	**0.010**
**Male newborn (ref)**				
Female newborn	55,028.2	66,889.7	0.82	0.414
**Weight at birth (per gram)**	− 334.6	104.4	−3.2	**0.002**
**APGAR at the fifth min (per point)**	20,287.7	21,584.7	0.94	0.351
**Ubudehe Category 1 (ref)**				
Ubudehe Category 2	22,777.5	103,600.9	0.22	0.826
Ubudehe Category 3	119,325.8	101,747.2	1.17	0.246
Ubudehe Category 4	− 145,912.5	219,607.7	−0.66	0.509
**Delivery by cesarean section (ref)**				
Delivery mode-Normal delivery	40,675.9	45,060.4	0.902	0.368
R Square	**R**^**2**^ **= 0,613**			

Dependent Variables: Total MDC, *CA* Catchment area, *HC* Health Center

### Factors predicting MDC

As shown in Table 6, BW was the most significant predictive factor of MDC of initial hospitalization of PBs. Other predictive factors included hospital category, oxygen therapy, and being insured by Rwanda Social Security Board (RSSB). The latter is linked to differences in unit costs. Birth weight was the significant negative predictive factor while others are positive predictive factors. This means that MDC progressively and discretely decreases as the BW increases. It is worthy to note that the variables in Table 5 explain almost 60% of the MDC of initial hospitalization of a PB and that BW alone explains almost 30% of the total medical direct costs. PBs with similar underlying conditions were usually treated in a similar way, and there were no patients with zero cost. Hence the data do not deviate much from the requirements of OLS for the residuals to be approximately normally distributed and with constant variance.

## Discussion

This study explored medical direct costs (MDC) associated with the initial hospitalization of premature babies (PBs) in Rwanda. To the best of our knowledge, this is the first time that the MDC of initial hospitalization of PBs in Rwanda has been determined in different hospitals across the country in a way that allows the comparison of costs at different levels of care. The characteristics of the PBs and their mothers included in the study are discussed first followed by sections examining the significance of the MDC analysis.

### Characteristics of the premature babies and mothers

Our results indicate that moderate and late preterm births account for the major proportion of all PBs (63.4%) followed by very PBs (36.6%). These results corroborate with those of Zainal et al. in Malaysia whose findings revealed that the majority of PBs were also from moderate and late preterm groups (59%) followed by very preterm groups (32%) [13].

The median BW of the PBs was 1800 g which is higher than that found by Akman et al. in Turkey (1560 g [16]. The average BW (1724 g) is also higher than of Comert et al. in Turkey (1689 g), though the median gestational ages in the two studies are almost similar. The differences in BW could be attributed to the level of care of the study hospitals, since most of our hospitals were district hospitals while hospitals for the other 2 studies were tertiary hospitals.

For the clinical features of PBs, 60% had respiratory distress syndrome (RDS) as found by other studies [12, 13, 16]. This has an impact on the costs since RDS increases oxygen use. Any intervention aiming at reducing RDS should be compared against its cost-benefit and be altered if cost-effective. The average LoS was 14.6 days corroborating with findings of Desgualdo in Brazil and Comert in Turkey who found an average LoS of 14 and 13.6 days respectively [12, 14].

The majority of mothers (67, 4%) were in low socioeconomic classes according to the categories used in Rwanda [23]. This suggests that the cost of initial hospitalization of prematurity is more costly for families in lower socioeconomic classes. It is very worthwhile to indicate that almost all mothers had health insurance which improves their access to health care and provides financial protection against possible catastrophic medical expenditures for the family.

Most of the babies were born to mothers who had not taken corticosteroids when indicated. This could be attributed to the fact that most of the mothers consult the health care providers a bit late when they are ready to deliver so that corticosteroids are judged no longer useful. However, health facilities should always ensure that they have stock available of this drug to prescribe it according to the guidelines. The enhancement of corticosteroids use would greatly lower the incidence of RDS and thus oxygen use, which is such a substantial factor in MDC.

### Average medical direct costs and costs per category

Our findings revealed that the average MDC for treating one PB was 237.7 USD with the oxygen-related cost amounting to 38.4% of the total MDC and a daily cost of $16.4. Taking into account the country’s Gross Domestic Product (GDP) per capita per year which is $845 in Rwanda, the average MDC for the initial hospitalization of a PB is equivalent to 28% of GDP per capita [26, 27]. These costs may impact the general economy of Rwanda and may be catastrophic out of pocket expenditures for a person not covered by insurance. Thus, timely preventive interventions to reduce preterm birth rate like systematic urinary tract infection diagnosis and treatment during antenatal care, improved mother’s nutrition and anemia prevention among pregnant women might be cost-savings on hospital budget [28].

By comparing our costs-related results to those of the international literature, differences and similarities have been observed. A study conducted by Desgualdo et al. in 2004 in Brazil showed that the average MDC for initial hospitalization of a PB was $2386. By taking into account the GDP per capita in Brazil at the time of the study, which was $3637, this average MDC was 65.6% of GDP per capita per year (https://data.worldbank.org/indicator/NY.GDP.PCAP.CD). Most of the difference could be attributable to the fact that the Brazilian study was carried out only in a tertiary hospital, although other factors may contribute to the explanation.

Our results are not very different from the study conducted by Comert et al. in Turkey which revealed that the mean total hospitalization cost of a PB was $4187 [14]. This mean represented 35% of GDP per capita per year, estimated to be $11,795 in Turkey in 2012 (https://data.worldbank.org/indicator/NY.GDP.PCAP.CD).

Also, our results are comparable with that of a study conducted in Greece by Geitona et al. who determined the average cost per preterm baby during the initial hospitalization as $6801 [15], representing 24% of total GDP per capita per year which was $28,827.3 in 2007 (https://data.worldbank.org/indicator/NY.GDP.PCAP.CD).

However, in India, Naranga et al. calculated $125 as the mean cost per day of hospitalization which is estimated to be 17.5% of the GDP per capita per year in India in 2005 [17]. A cost which is very high compared to the average cost per day of $16.4 in Rwanda, representing 2% of total GDP per capita during the year of study. The fact that the costs in Rwanda seem to be a bit lower than many countries, apart from Greece, may be related to the subsidization of health care for newborns by the Government of Rwanda in its efforts toward Universal Health Coverage (UHC).

Furthermore, the reasons accounting for differences in costs across different studies and countries may be multiple and may include the time at which the study was conducted, inflation rates, variations in medical practices, health financing mechanisms, study settings and unit cost per item per country among others.

Finally, oxygen cost was found to account for the high proportion of total MDC. This is a result unique to our investigation and not found in any other study among the ones reviewed. This could be attributable to the mechanism of production and supply of oxygen in Rwanda where pressurized gas cylinders are the most common form of oxygen storage used in hospitals. The oxygen is produced in a manufacturing plant and is stored as compressed gas in a cylinder. Consequently, transportation to and from the private manufacturing facility is required and the cost of procuring these cylinders includes the return on investment for the private manufacturers. We think that the unit cost of oxygen could be reduced by locating an oxygen generation system within the hospitals. This can be done by equipping hospitals with an oxygen plant to allow the production of oxygen onsite from ambient air. Though this is associated with a high initial cost of installing the plant, the authors show that hospitals sustain the lowest annual costs when using the on-site oxygen production scenario in long run [29].

### Medical direct costs per level of hospital, level of prematurity and weight category

Our results revealed that the average MDC varies with the weight and age at birth, a result confirmed by previous studies [12–14, 16, 30, 31]. However, we were not expecting to have the average MDC of the regional referral hospital higher than that of a University Teaching Hospital. The average weight is almost the same for the 2 hospitals, but the LoS is almost double for the regional referral hospital. Explicative factors of this difference may include the fact that the regional referral hospital is a new structure with new and young specialists with insufficient capacity for diagnosis. These two factors may allow variation in medical practice where new specialists may use expensive drugs when they are uncertain of the type of care and will extend the LoS. The University Teaching Hospital will have experienced staff with more effective diagnostic skills and equipment with the example of advanced laboratories allowing them to use more cost-effective means of treatment including best practice selection of drugs and thus lowering the LoS.

### Costs category not covered by insurance schemes

Our results indicated almost 13% of MDC is not covered by insurance. The costs not covered included half of the total costs related to professional services, some key drugs, use of continuous airway pressure (CPAP) machines and incubators. It is likely that paying these critical items may cause out of pocket catastrophic expenditure in some cases as they are added to the co-payment and in most cases, the patient has no alternative.

### Medical direct costs predictive factors

Birth weight is the most powerful predictive factor for MDC. This was also confirmed by other studies [14, 16, 30, 31]. This information is helpful to provide the estimate of MDC as well as introducing quantitative information for health planning and resource allocation for PTB in the country. This information is helpful while planning interventions to reduce PTB by comparing the costs of interventions against the MDC of hospitalizing a PB.

### Implications and future research

Our findings have implications for prematurity prevention and management. The results indicated that some MDC are not reimbursed by the insurance schemes with a risk of being out of pocket catastrophic expenses to insured people. These include drugs like prophylactic aminophylline and caffeine, shown to be effective in reducing apnea incidence in higher-risk neonates, and fercefol used to prevent anemia [32, 33]. Some key interventions including using incubators and continuous positive airway pressure are also not covered. The study recommends the revision of the coverage list to include all high priorities and essential drugs and ensure critical items are part of the entitlements (benefits) in the neonatal unit. More importantly, the cost of these interventions and drugs may be offset by the reduction of the costs of other drugs, professional services, and hospitalization-related costs. In addition, we found that the cost of oxygen is very high, so there is a need to analyse the cost of life-saving drugs like surfactants against its cost-effectiveness and consider them based on the evidence. The oxygen generation system may also reduce the oxygen-related costs. Furthermore, the costs of interventions to reduce prematurity should be examined as a means to prevent high MDC for the PBs since the overall cost of prematurity is high (28% of GDP per capita per year). Our results also revealed that some unit costs are different depending on the insurance scheme within the same hospital and we recommend that unit costs should be standardized and that the difference between the standardized cost and the insurance coverage could be accounted for in the form of co-payment if possible.

Additional research could be considered, including a study to examine the indirect costs associated with initial hospitalization of premature babies as well as costs incurred post-hospitalization (i.e. the impact on families, informal carers, and the work productivity of parents). Further research is warranted to detect the possible relationship between variations in medical practice within neonatal departments and differences in MDC of initial hospitalization of premature babies across hospitals.

### Strength and limitations of the study

The strengths of the study included the fact that it was conducted prospectively and that it included all types of public hospitals in Rwanda. Our study is unique for Rwanda but adds to a growing body of international literature. However, comparison between costs in different countries must be done cautiously, taking into account mediating factors including the years of study, different price levels, patient co-payments, and different means of reimbursing suppliers, among others. The study has some potential limitations. First, if a PB was referred from one facility to another, the total cost should be estimated for the entire treatment period until the PB is finally discharged from the last hospital. Unfortunately, our study did not consider the cost beyond the study hospital. This might underestimate the total overall costs. Second, it might have been better to include more than one regional referral hospital and more than one tertiary hospital as was done for district hospitals. This would have given a better insight into the underlying reasons for the cost difference between tertiary and regional referral hospitals. Third, OLS requires cost data are normally distributed, though is usually robust to deviations from this assumption. Finally, the study did not include private hospitals, where unit costs are thought to be greater than public hospitals. However, the comparison between private and public costs may be misleading. Hospitals in Rwanda are predominantly public and very few PBs are admitted to private hospitals which are concentrated in the capital city. Furthermore, public hospitals have well-equipped neonatal services, and most critically ill PBs are treated there, while private hospitals are generally able to treat only those in stable conditions. Most people are covered by CBHI which does not cover them in private hospitals.

## Conclusion

The primary purpose of the current study was to fill an important gap in knowledge by examining the MDC associated with the initial hospitalization of prematurity in Rwanda. Birth weight was shown to be the most powerful predictor of MDC. These results can contribute to evidence-based planning of interventions to reduce preterm birth and also effective resource allocation to manage prematurity.

## Supplementary Information

**Additional file 1: Supplementary file 1.** Overall average and per hospital category, length of stay, weight and age at birth. **Supplementary file 2.** Socioeconomic and demographic characteristics of mothers. **Supplementary file 3.** Clinical features and length of stay per category.

## Data Availability

The datasets used in this study may be available from the corresponding author upon a reasonable request.

## Abbreviations

APGAR: Appearance, Pulse, Grimace, Activity, Respiration
BW: Birth Weight
CBHI: Community Based Health Insurance Scheme
CMHS-IRB: College of Medicine and Health Sciences Institutional Review Board
COI: Cost Of Illness
CPAP: Continuous Positive Airway Pressure
GDP: Gross Domestic Product
ICU: Intensive Care Unit
LoS: Length of Stay
MDC: Medical Direct Costs
PBs: Premature Babies
PTB: Preterm Birth
PI: Principal Investigator
RAMA: La Rwandaise d’assurance Maladie
RDS: Respiratory Distress Syndrome
RNEC: Rwanda National Ethics Committee
RSSB: Rwanda Social Security Board
