# Supplementary material for: Evaluating the medical direct costs associated with prematurity during the initial hospitalization in Rwanda: a prevalence based cost of illness study
Source: BMC Health Serv Res. 2022 Jul 27;22:953. doi: 10.1186/s12913-022-08283-w (PMC9327227; doi:10.1186/s12913-022-08283-w)
Supplement: Supplementary file 1 — Additional file 1: Supplementary file 1. Overall average and per hospital category, length of stay, weight and age at birth. Supplementary file 2. Socioeconomic and demographic characteristics of mothers. Supplementary file 3. Clinical features and length of stay per category. [file 12913_2022_8283_MOESM1_ESM.docx]

**Supplementary file 1: Overall average and per hospital category, length of stay, weight and age at birth**

| **Variable** | **Hospitals** | **Mean** | **Minimum** | **Maximum** | **Median** | **SDV** |
| --- | --- | --- | --- | --- | --- | --- |
| **LoS (days)** | All hospitals | 14.7 | 2 | 67 | 9 | 13.6 |
| **LoS per Hospital category** | DHs | 11.5 | 2 | 67 | 7 | 11.7 |
|  | Regional Ref | **25** | **5** | 62 | 18 | 15.8 |
|  | Tertiary | 14 | 3 | 40 | 12 | 10.29 |
| **Weight** | All hospitals | 1724 | 900 | 2970 | 1800 | 408.1 |
| **Weight per hospital category** | DHs | 1811 | 950 | 2970 | 1800 | 400.0 |
|  | Regional Ref | 1587 | 900 | 2500 | 1700 | 423.2 |
|  | Tertiary | 1591 | 1170 | 2240 | 1500 | 322.7 |
| **Age at birth** | All hospitals | 32W+3D | 28W | 36W+5D | 32W+5D | 2W+2D |
| **Age per hospital category** | DHs | 32W+6D | 28W | 36W+5D | 33W | 2W+3D |
|  | Regional Ref | 32W+2D | 29W | 36W+1D | 32W | 2W+1D |
|  | Tertiary | 31W+1D | 28W | 35W | 30W | 2W+3D |

DHs= District Hospitals, Ref=Referral , SDV: Standard Deviation, LOS: Length of Stay

**Supplementary file 2: Socioeconomic and demographic characteristics of mothers**

| **Variables** | **Variable category** | **Frequency** | **Percent** |
| --- | --- | --- | --- |
| **Socioeconomic category**  **(Ubudehe)** | category 1 | 19 | 15.4 |
|  | category 2 | 64 | 52.0 |
|  | category 3 | 37 | 30.1 |
|  | category 4 | 3 | 2.4 |
|  | **Total** | **123** | **100** |
| **Type of insurance** | CBHI | 114 | 92.7 |
|  | RAMA/RSSB | 4 | 3.3 |
|  | MMI | 1 | 0.8 |
|  | Other insurance | 3 | 2.4 |
|  | No insurance | 1 | 0.8 |
|  | **Total** | **123** | **100** |
| **Education** | None | 15 | 12.2 |
|  | Primary | 74 | 60.2 |
|  | Secondary | 26 | 21.1 |
|  | University | 8 | 6.5 |
|  | **Total** | **123** | **100.0** |
| **Age** | Below 20 years | 10 | 8.1 |
|  | 20-29 years | 57 | 46.3 |
|  | 30-39 years | 50 | 40.7 |
|  | 40 years and above | 6 | 4.9 |
|  | **Total** | **123** | **100.0** |
| **Number of**  **deliveries** | 1 | 44 | 35.8 |
|  | 2 | 23 | 18.7 |
|  | 3 | 23 | 18.7 |
|  | 4 and above | 33 | 26.8 |
|  | **Total** | **123** | **100** |

MMI= Military Medical insurance, RSSB= Rwanda Social Security Board, CBHI: Community Based Health Insurance (CHBI)

**Supplementary file 3 : Clinical features and length of stay per category**

| **Variable** | **Variable category** | **Frequency** | **Percent** |
| --- | --- | --- | --- |
| **Comorbidities** | RDS | 74 | 60.2 |
|  | Jaundice | 65 | 52.8 |
|  | Neonatal infection | 38 | 30.9 |
|  | Hypothermia | 38 | 30.9 |
|  | Cerebral anoxia | 33 | 26.8 |
|  | Hypotrophy | 23 | 18.7 |
| **Outcome** | Improved discharged | 106 | 86.2 |
|  | Referred | 3 | 2.4 |
|  | Died | 14 | 11.4 |
|  | Lost to follow up | 0 | 0.0 |
| **LoS for PBs** | <= 7 days | 52 | 42.3 |
|  | 8 to <15 days | 27 | 22.0 |
|  | 15 to < 22 days | 16 | 13.0 |
|  | 22 to 28 days | 12 | 9.8 |
|  | >28 days | 16 | 13.0 |

RDS: Respiratory distress syndrome
